# Effects of propofol versus thiopental on Apgar scores in newborns and peri-operative outcomes of women undergoing emergency cesarean section: a randomized clinical trial

**DOI:** 10.1186/s12871-015-0044-6

**Published:** 2015-04-29

**Authors:** Janat Tumukunde, Dlamini Diana Lomangisi, Ocen Davidson, Andrew Kintu, Ejoku Joseph, Arthur Kwizera

**Affiliations:** Department of Anesthesia, Makerere University College of Health Sciences, Mulago National Referral Hospital, P.O. Box 7051, Kampala, Uganda

**Keywords:** Propofol, Thiopental, General anesthesia, Cesarean section, Apgar score

## Abstract

**Background:**

General and regional anesthesia are the two main techniques used in cesarean section. Regional anesthesia is preferred, but under certain circumstances, such as by patient request and in patients with back deformities, general anesthesia is the only option. Commonly used induction agents include thiopental, ketamine, and propofol, depending on availability and the maternal clinical condition. The objective of this study was to investigate the effects of thiopental and propofol on the neonatal Apgar score and maternal recovery time following emergency cesarean section in order to determine the superior agent for mothers and neonates.

**Methods:**

This single-blinded randomized clinical trial included 150 ASA I and II patients block-randomized equally between the two study arms. Pregnant women at term scheduled to undergo cesarean section and their neonates were enrolled. The primary outcomes were the Apgar scores through 10-min postpartum, resuscitation requirement, and admission to the neonatal intensive care unit. The secondary outcome was the maternal recovery times.

**Results:**

At 0 min (umbilical cord clamp time), 43 (57.3%) neonates in the propofol group had an Apgar score < 7 compared with 31 (41.3%) neonates in the thiopental group (p = 0.05). The maternal recovery time was shorter in the propofol group than in the thiopental group (25 min vs. 31 min, respectively, p = 0.003).

**Conclusion:**

Apgar scores do not differ significantly whether thiopental or propofol is used for anesthetic induction in women undergoing general anesthesia for an emergency cesarean section.

**Trial registration:**

Pan-African Clinical Trial Registry (#PACTR201306000536344) http://www.pactr.org/ATMWeb/appmanager/atm/atmregistry?_nfpb=true&_pageLabel=atm_portal_page_mytrials

## Background

Regional anesthesia is generally preferred during cesarean section, but general anesthesia may be the only option under certain circumstances such as patient preference, back deformities not amenable to spinal anesthesia, failed spinal anesthesia, intracranial hypertension, maternal coagulopathy, and certain neurologic diseases [1]. A safe induction agent for obstetrics should, among other things, provide a smooth, quick induction, maintain maternal hemodynamic function, and exert minimal to no effect on the Apgar score. Thiopental has been routinely used as an anesthetic induction agent for cesarean section since the 1930s and is the standard against which all new agents are compared. However, it has several disadvantages, including decreased maternal arterial pressure, which, when coupled with a long induction time, can reduce the Apgar score [2]. Propofol is widely used for induction and maintenance of anesthesia in other surgeries but not in obstetric procedures. It has a short induction time and blunts airway reflexes during laryngoscopy; compared with thiopental, patients emerge faster from propofol anesthesia [3]. However, propofol also crosses the placenta [4,5] and thus, can depress the fetal central nervous system, resulting in a low Apgar score at birth [6,7].

The population of pregnant mothers requiring cesarean section either by default or by request is rising, [8] and the lack of skilled labor in Uganda is a constant problem [9]. Maternal and child health is the focus of millennial development goals 4 and 5, and improving safety and reducing maternal and neonatal mortality are necessary to achieve these goals. Therefore, the objective of this study was to investigate the effects of thiopental and propofol on the neonatal Apgar score and maternal recovery time following cesarean section.

## Methods

### Study design and setting

This randomized single-blinded clinical trial was performed at Mulago Hospital, which is the main national referral hospital in Uganda. Its obstetric department receives mothers from Kampala and the surrounding region. Approximately 30,000 deliveries are performed annually and 600 cesarean sections monthly, of which 15% are performed under general anesthesia. The high rate of general anesthesia for cesarean sections is due to the erratic availability of spinal needles and drugs, and unfounded patient fears over lumbar punctures in general. Anesthesia is performed by anesthesiologists, anesthetic officers, or postgraduate students in anesthesia.

### Study population

We included ASA class I and II term pregnant mothers scheduled to receive general anesthesia for an emergency cesarean section and excluded all patients potentially allergic to propofol or thiopental.

### Randomization, blinding, and enrollment

Participants were block randomized into the two study arms. An independent statistician randomly generated the sequence of participant allocation to the thiopental or propofol treatment groups. First, random blocks of 4–10 participants were generated, and within each block, a random sequence for the participant intervention groups was generated and labeled either 1 (one) for propofol or 2 (two) for thiopental. This sequence was concealed from all participants by inserting it into opaque, sequentially arranged sealed envelopes. The intervention group allocations were placed in small envelopes that were then placed in bigger envelopes representing the blocks. At the time of participant recruitment, the study investigator retrieved the next available small envelope, which indicated the intervention group, from the next available block envelope and handed it to the participant.

The patient, midwife, and pediatrician were blinded to the group assignment, but the anesthesia provider was not. The anesthesia providers were either qualified anesthesiologists or residents in their final year of residency. After screening for inclusion and exclusion criteria, eligible women were consecutively enrolled in the study.

### Sample size

This was a non-inferiority study. To determine whether a 20% difference in the Apgar score existed between thiopental and propofol treatment, 150 patients were required to be 90% certain that the upper limit of a one-sided 95% confidence interval (or a 90% two-sided interval) excluded a greater than 20% difference in favor of the standard (thiopental) group.

### Intervention and anesthetic technique

Each patient was wheeled into the surgical suite in a left lateral position and transferred to the operating table, and a wedge was placed under the right hip to achieve a leftward uterine displacement of 15°. Routine monitors were attached (pulse oximeter for heart rate and oxygen saturation, continuous ECG monitor, and automatic blood pressure monitor). A wide-bore intravenous cannula (16- or 18-G) was placed in the less dominant forearm for administration of drugs and fluids. Intravenous metoclopramide (10 mg) and ranitidine (50 mg) diluted in 20 ml of saline was administered over 2 min. The surgical site was then scrubbed and draped aseptically by the surgeon. Denitrogenification with 100% oxygen was performed for 3 min, and lignocaine (1–2 mg/kg) was administered to blunt the laryngeal reflexes and numb the catheterized vein to the nociceptive effects of the induction drugs. The induction agent was administered, either propofol (2 mg/kg) or thiopentone (4 mg/kg) depending on which study arm the patient was enrolled in, cricoid pressure was applied, and suxamethonium (1.5 mg/kg) administered. Using a laryngoscope, tracheal intubation was performed, confirmed, and secured within 2 min. The patient was maintained on isoflurane (1–1.5%) mixed with 100% oxygen flowing at 2.5 L/min until the start of skin closure. Nitrous oxide and ambient air were not used as these agents are unavailable at this hospital.

Oxytocin (5 IU) diluted in 5 ml of saline was administered as a slow bolus after delivering the baby’s shoulders. Pain was controlled by administering 0.1 mg/kg of morphine after the baby’s umbilical cord was clamped. Rectal paracetamol was also administered at the end of the procedure. All patients received 1,500 ml of crystalloids (normal saline or lactated Ringer’s solution). If hypotension, which was defined as a 20% reduction from the baseline blood pressure, was encountered, then additional crystalloids and vasopressors were administered at the discretion of the anesthesia provider.

### Primary outcomes

The neonatal Apgar score was assessed and recorded at 0, 1, 5, and 10 min after the umbilical cord was clamped by the midwife. The requirement for resuscitation and admission into the neonatal special care unit (NICU) was also recorded by the study assistant. Criteria for NICU admission was any neonate requiring more than 3 min of continuous bag mask ventilation during resuscitation as per NICU protocols.

### Secondary outcomes

The maternal recovery time was measured, defined as the duration from induction to complete orientation in time, place, and person.

### Data management

All data were double entered into Epidata version 3.1 software, with range, consistency, and validation checks embedded to aid data cleaning. The data were analyzed using Stata version 12 software.

Categorical data, including the number of participants and the respective proportions, are presented in tabular, graphical and text forms, and categorized into propofol and thiopental groups. The two groups were compared using the Chi square test. Continuous data are presented as the mean with standard deviation, and were compared between the groups using the *t*-test to detect any significant differences. The median and interquartile range of the decision-to-delivery interval was calculated, and the differences between the groups were determined using non-parametric testing. A p-value of < 0.05 indicated statistical significance.

An intention-to-treat analysis method was used, and the main outcome of interest was an Apgar score of less than 7. The Poisson regression model was used to assess the incidence risk of the outcome between the two randomization groups and to estimate the risk ratio and confidence interval. Baseline characteristics were equally distributed between the randomization groups, and none of the outcomes differed significantly between the randomization groups. Therefore, multivariate analysis for potential confounders was not performed.

### Data safety management board

The Data Safety Management Board (DSMB) comprised an anesthesiologist, statistician, obstetrician, and a pharmacist who were informed if an adverse event occurred. The study would be discontinued if 10% of study subjects in one study arm experienced an adverse event associated with the study drugs as determined by the DSMB, or if a p value of <0.025 was obtained on an interim analysis performed 3 months after beginning the study.

### Ethical considerations

This study was approved by the Department of Anesthesiology, Mulago Hospital Ethics Committee, the School of Medicine Research and Ethics Committee (SOMREC), and the Department of Obstetrics and Gynecology. All patients provided informed, written consent before the start of surgery. This trial is registered in the Pan-African Clinical Trial Registry (#PACTR201306000536344).

## Results

**Figure 1:**
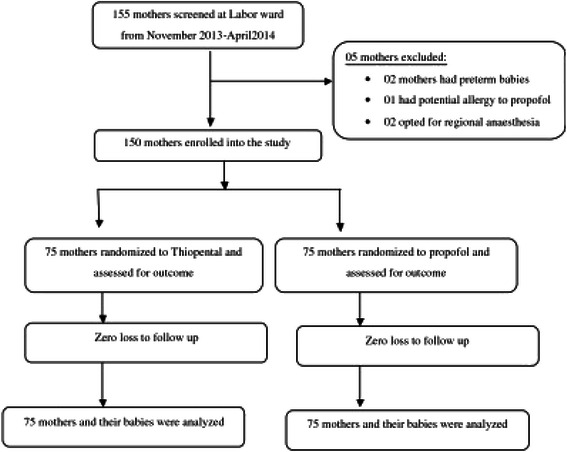
Summary of patient recruitment and allocation.

A total 150 pregnant women were enrolled from November 2013 to April 2014 as shown in Figure 1.

### Maternal baseline characteristics

Maternal baseline characteristics were equally distributed between the two study groups. The mean maternal age in the propofol and thiopental groups was 25.2 years and 24.2 years, and the mean weight was 68.6 kg and 66.7 kg, respectively. The median overall decision-to-delivery interval was 137.5 (IQR 20–968), and there was no statistically significant difference between the median intervals in the propofol (median 145; IQR 20–600) and thiopental (median 120; IQR 26–900; p = 0.540) groups. The induction-to-delivery interval and the surgical duration also showed no significant differences between the groups (Table 1).

**Table 1:** **Maternal baseline characteristics**

Variable	Overall	Propofol group	Thiopental group
**Maternal age**	24.67 (5.64)	25.16 (5.11)	24.17 (6.11)
**Maternal weight**	67.61 (9.51)	68.56 (9.31)	66.67 (9.68)
**Decision-to-delivery interval (min)**	212.52 (218.45)	220.99 (213.04)	204.05 (224.84)
**Induction-to-cord clamping interval (min)**	8.52 (4.48)	8.92 (5.01)	8.12 (4.47)
**Surgical duration (min)**	38.7 (13.4)	38.9 (12.5)	38.1 (14.9)

Data are presented as the mean (standard deviation).

### Apgar score distribution

A total 74 neonates had an Apgar score < 7. Of those, 43 were in the propofol group and 31 in the thiopental group, with an incidence ratio of 1.42 (95% CI = 0.88–2.32) and a p-value of 0.068.

At 0 min, 43 (57.3%) neonates in the propofol group had an Apgar score < 7, compared with 31 (41.3%) neonates in the thiopental group. At 1 min, 35 (46.7%) and 24 (32%) neonates had an Apgar score <7 in the propofol and thiopental groups, respectively. At the 5-min mark, 13 (17.3%) and 8 (10.7%) neonates had Apgar scores < 7 in the propofol and thiopental groups, respectively, while at the 10-min mark, 3 (4%) neonates in the propofol group and 2 (2.7%) in the thiopental group had an Apgar score < 7.

**Figure 2:**
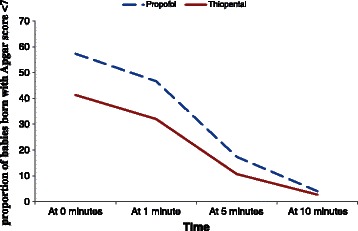
Percentage of neonates with a postpartum Apgar score < 7.

The incidence of neonates with an Apgar score < 7 at each interval is summarized in Table 2. There were no statistically significant differences at any of the time points, except at time 0, which had a marginally significant difference (p = 0.05). The proportion of neonates with an Apgar score < 7 gradually decreased and did not differ significantly between the groups at 10 min. The trends are further detailed in Figure 2.

**Table 2:** **Percentage of neonates with an Apgar score < 7 post-cesarean section**

Apgar score	Propofol group	Thiopental group	% difference (95% CI)	p-value
**0 min**	43 (57.33)	31 (41.33)	16.00 (0.2–31.80)	0.050
**1 min**	35 (46.67)	24 (32)	14.67 (-0.7–30.12)	0.066
**5 min**	13 (17.33)	8 (10.67)	6.67 (-4.39–17.72)	0.239
**10 min**	3 (4)	2 (2.67)	1.33 (-4.41–7.07)	0.649

Data are presented as the number (percentage) of patients. CI, confidence interval.

### Neonatal intervention and NICU admission

Maneuvers to improve Apgar scores included gentle stimulation or active resuscitation, which could be brief or prolonged. In total, 14 of 74 neonates born with an Apgar score < 7 required gentle stimulation to raise the Apgar score > 7. Of these, seven were in the propofol group and seven in the thiopental group. The remaining 60 neonates required active resuscitation including stimulation, bag mask ventilation, Laryngeal Mask Airway or endotracheal tube insertion, and ventilation, which was discontinued once the neonate demonstrated adequate spontaneous respiration. A total 19 (52.8%) neonates with an Apgar score ≤ 7 recovered (improved Apgar scores) after < 5 min of resuscitation in the propofol group compared with 12 (50%) neonates in the thiopental group. Additionally, 1 (2.78%) neonate in the propofol group and 2 (8.33%) in the thiopental group died. As per protocol, any neonates with low Apgar scores who required continuous bag mask ventilation for >3 min were admitted for close observation for at least 24 hours.

A total 22 neonates were admitted into the NICU for close observation, comprising 16 (21.33%) neonates from the propofol group and 6 (8%) from the thiopental group. Of these, 2 (13.3%) neonates in the propofol group continued to have an Apgar score < 7 (Table 3).

**Table 3:** **Neonatal resuscitation**

Variable	Propofol	Thiopental	Overall	p value
**Active resuscitation**				
**No**	39 (52.00)	51 (68.00)	90 (60.00)	
**Yes**	36 (48.00)	24 (32.00)	60 (40.00)	0.046
**Time to recovery (min)** ^**a**^				
**<5**	19 (52.78)	12 (50.00)	31 (51.67)	
**5–10**	14 (38.89)	10 (41.67)	24 (40.00)	
**Died**	1 (2.78)	2 (8.33)	3 (5.00)	
**Apgar score < 7**	2 (5.56)	0	2 (3.33)	0.518
**NICU admission**				
**No**	59 (78.67)	69 (92.00)	128 (85.33)	
**Yes**	16 (21.33)	6 (8.00)	22 (14.67)	0.021
**Apgar score at admission** ^**b**^				
**<7**	2 (13.33)	0	2 (0.09)	
**≥7**	14 (86.67)	6 (100)	20 (95.45)	0.531

Data presented as the number (percentage) of neonates.

^a^Measured only in neonates who were resuscitated.

^b^Measured only in neonates admitted to the neonatal intensive care unit (NICU).

### Maternal recovery time

The mean recovery time was significantly shorter in the propofol group (25.1 min) than in the thiopental group (31.4 min; p = 0.003).

## Discussion

We conducted a single blinded randomized clinical trial comparing the effects of propofol and thiopental on neonatal Apgar scores and maternal peri-operative outcomes.

The Apgar scores of neonates randomized into the two study arms did not significantly differ, except at 0 min, which showed a marginally significant difference. However, the percentage of neonates with unsatisfactory scores (<7) was greater than 50% in the propofol group (57.3%) compared with that in the thiopental group (41.3%). While this may not be statistically significant, this outcome cannot be ignored because it highlights the need for skilled labor to attend all births when anesthesia is induced with propofol. The high prevalence of unsatisfactory Apgar scores can be attributed to the high rate of placental transfer of propofol coupled with the rapid loss of consciousness, as reported in previous studies [5,10].

The percentage of neonates with an Apgar score < 7 at the 1, 5, and 10-min intervals steadily decreased in both study groups to nearly equal numbers of three and two neonates in the propofol and thiopental groups, respectively (Figure 2). None of the differences in the Apgar scores were significant between the two groups, except at 0 min, which showed borderline significance, despite the Apgar scores being higher in the propofol group than in the thiopental group (Table 2). This finding is consistent with those of other studies comparing these two induction agents [11-13]. The faster improvement in the Apgar scores in the propofol group is probably caused by the fast redistribution half-life of propofol, which is as low as 1 min [14-16], while thiopental requires up to 6 min [17]. This also explains the higher percentage of neonates who recovered in <5 min in the propofol group.

An important finding in our study was the significantly higher rate of NICU admissions in the propofol group compared with the thiopental group. All but three neonates admitted to the NICU survived at 24 hours.

The three neonates that died in this study had low Apgar scores coupled with poor respiratory effort; they required ventilator assistance, but because these services were unavailable in the NICU, they died. A large percentage of neonates were admitted into the NICU (22% overall) not only because of low Apgar scores, but also due to protocol. All neonates with low Apgar scores requiring continuous bag mask ventilation for >3 min are admitted for close observation for at least 24 hours.

The mean recovery times were significantly shorter in the propofol group than in the thiopental group at 25 min (10.13) versus 31 min (14.66), respectively (p = 0.003). Because the mean duration of isoflurane exposure was identical in the groups (38.9 vs. 38.1 min), the prolonged recovery time in the thiopental group can be solely attributed to the induction agent. Contribution from the initial maternal physiologic state cannot be discounted; however, under normal circumstances, this factor should be evenly distributed between the two study groups as the study population was randomized.

Our study had a number of limitations. This was a single-blinded study because of the consistency of the drugs evaluated; propofol is a white, milky suspension, and thiopental is a yellow, clear solution. The anesthesia providers are aware of this difference; therefore, blinding them is impossible. Any individual variation in the metabolism of the induction drugs could not be measured; thus, this potential contribution to the low Apgar scores could not be accounted for. Finally, the interval between the decision to perform cesarean section and the delivery was significantly higher than the recommended 30 min for an emergency case, which also contributed to the low Apgar scores overall.

## Conclusions

In conclusion, the Apgar scores did not differ significantly whether thiopental or propofol was used as an induction agent in women receiving general anesthetic for an emergency cesarean section. However, there was a higher rate of NICU admission among neonates in the propofol group. Propofol does offer the advantage of a shorter recovery time. In a referral center where cesarean sections under general anesthesia are inevitable whether by design or default, it is important to carefully select the induction agent. Furthermore, skilled personnel are required to attend to neonates delivered by cesarean section under general anesthesia.
